# Effects of Sample Size on Plant Single-Cell RNA Profiling

**DOI:** 10.3390/cimb43030119

**Published:** 2021-10-20

**Authors:** Hongyu Chen, Yang Lv, Xinxin Yin, Xi Chen, Qinjie Chu, Qian-Hao Zhu, Longjiang Fan, Longbiao Guo

**Affiliations:** 1 Institute of Crop Science and Institute of Bioinformatics, Zhejiang University, Hangzhou 310027, China; hongyuchen@zju.edu.cn (H.C.); yinxx0416@zju.edu.cn (X.Y.); xich@zju.edu.cn (X.C.); qinjiechu@zju.edu.cn (Q.C.); fanlj@zju.edu.cn (L.F.); 2 State Key Laboratory of Rice Biology, China National Rice Research Institute, Hangzhou 310006, China; lvyang0725@163.com; 3 Rice Research Institute, Shenyang Agricultural University, Shenyang 110866, China; 4 CSIRO Agriculture and Food, Black Mountain Laboratory, GPO Box 1700, Canberra, ACT 2601, Australia; Qianhao.Zhu@csiro.au; 5 School of Medicine, Zhejiang University City College, Hangzhou 310027, China

**Keywords:** single-cell RNA (scRNA), cell number, sampling coverage, *Arabidopsis thaliana*

## Abstract

Single-cell RNA (scRNA) profiling or scRNA-sequencing (scRNA-seq) makes it possible to parallelly investigate diverse molecular features of multiple types of cells in a given plant tissue and discover cell developmental processes. In this study, we evaluated the effects of sample size (i.e., cell number) on the outcome of single-cell transcriptome analysis by sampling different numbers of cells from a pool of ~57,000 *Arabidopsis thaliana* root cells integrated from five published studies. Our results indicated that the most significant principal components could be achieved when 20,000–30,000 cells were sampled, a relatively high reliability of cell clustering could be achieved by using ~20,000 cells with little further improvement by using more cells, 96% of the differentially expressed genes could be successfully identified with no more than 20,000 cells, and a relatively stable pseudotime could be estimated in the subsample with 5000 cells. Finally, our results provide a general guide for optimizing sample size to be used in plant scRNA-seq studies.

## 1. Introduction

Cells in biological tissues usually exhibit heterogeneity in morphology, signaling status, and genotype [1]. Traditional RNA-sequencing methods use samples with multiple types of cells and estimate the expression of bulked cells in a tissue as an average value, ignoring the diversities of different cell types. Single-cell RNA-sequencing (scRNA-seq)—a rapidly developing technology—makes it possible to perform molecular studies in parallel on multiple types of cells in a given tissue and promotes elucidation of cellular heterogeneity and discovery of developmental processes underpinning cell differentiation [2]. Single-cell transcriptomic analysis providing direct indicators of cell status has been widely used in multiple animal models to dissect cell heterogeneity. For example, the intratumoral heterogeneity in many types of cancer has been thoroughly studied by single-cell RNA profiling [3,4,5,6].

The first deep-sequencing analysis of single-cell transcriptome was reported in 2009 [7]. In the past decade, a variety of scRNA-seq methods have been developed to effectively amplify and enrich transcripts from single cells, including Smart-seq2 [8], STRT-seq [9], CEL-seq2 [10], and Drop-seq [11]. Smart-seq2 is a full-length transcript sequencing solution that enables investigation of long noncoding RNAs with poly(A) tail and alternative splicing events [8]. One of the most used scRNA-seq methods, 10× Genomics, combines microfluidic cell capture and Droplet barcoding, which allows sequencing thousands of single cells simultaneously and provides a high-throughput solution. As large amounts of input single cells can provide comprehensive biological information, 10× Genomics has been widely applied in human and animal scRNA-seq studies. It has been successfully applied to sequence transcripts in half a million nerve cells to create a detailed census of cell types in the mouse nervous system [12]. The cell types of each lobe of adult mouse cerebellum have been defined at the molecular level based on scRNA-seq data generated from more than 600,000 cells [13]. scRNA-seq data generated from large number of single cells enabled construction of cell type map of the tissue. Large number of cells have also been used in the investigation of transcription changes in tumors. For instance, in a study aiming for identification of cellular identities and compositions of human kidney tumors, transcriptomes of 72,501 cells collected from normal and diseased kidneys were sequenced using the scRNA-seq approach [14]. The more cells used in sequencing, the more biological information that could be generated; however, considering the cost involved in sequencing a huge number of cells, at the same time, processing a large number of single-cell data still is a very challenging task. Thus, it is necessary to take appropriate cell numbers into account according to the purpose of the studies.

Similar to tissues of humans and animals, plant tissues and cells are highly specialized not only morphologically but also biochemically and physiologically [15]. Plant cells usually have a hard outer structure, or cell wall, that complicates the separation of single cells [16]. Study at the single-cell level in plants is still in its early stages. Plant scRNA-seq is currently mainly focusing on certain tissues, from which protoplasts can be relatively easily isolated. The first plant scRNA-seq study was reported in 2015; it used 31 quiescent center (QC) and center column cells from *Arabidopsis* roots [17]. Later, scRNA-seq was scaled up to hundreds of cells in *Arabidopsis* [18] and maize [19] to investigate the cell types involved in root regeneration and differentiation of germinal cells, respectively. These studies achieved the outcomes that could not be fulfilled by using traditional transcriptome sequencing. With application of 10× Genomics technology in plants, the histological and cellular heterogeneity of *Arabidopsis*, rice [20], and maize [21,22,23] have been studied in detail. Especially, in 2019, five scRNA-seq studies on *Arabidopsis thaliana* root were published, each using ~3000 to ~12,000 cells [24,25,26,27,28]. Interestingly, although the number of cell clusters and their annotations varied amongst these studies, the common cell types (e.g., root hair cells, cortex, and endodermis) of *Arabidopsis* roots were all successfully identified by the five studies. While a large number of cells is required to resolve the structure of heterogeneous samples with many subpopulations, especially for the samples containing rare cell types [29], the relationship between the input cell numbers and the accuracy and sensitivity for uncovering certain subpopulations and/or rare cell types using scRNA-seq is still unclear. 

Two bioinformatics tools, Howmanycell (https://satijalab.org/howmanycells accessed on 29 August 2021) and SCOPIT [30] (www.navinlab.com/SCOPIT accessed on 29 August 2021), have been developed to estimate the appropriate number of cells to be sequenced in a specific single-cell study. The main input parameters of these prediction tools are the number of cell types, the proportion of rare cell types, and the expected minimum numbers of the targeted cell type to be identified. Both tools make a statistical estimation for the number of cells to be sequenced based on prior knowledge on the composition of the samples, but do not provide information on how sample size affects the performance of the tools and scRNA-seq results. 

In order to have an overview of the effect of sample size on plant scRNA-seq outcomes, in this study, we simulated and systematically compared the effects of sample coverage on downstream scRNA-seq analysis by sampling a different size of cells from a pool of ~57,000 *Arabidopsis thaliana* root cells that were investigated in the five studies mentioned.

## 2. Materials and Methods

### 2.1. Data Overview

Data of five—including four processed and one unprocessed—scRNA-seq experiments were used in this study. The four processed scRNA-seq datasets of *Arabidopsis* roots were retrieved from Gene Expression Omnibus (GEO; https://www.ncbi.nlm.nih.gov/gds accessed on 29 August 2021) under the accession numbers GSE121619, GSE122687, GSE123013, and GSE123818. GSE121619 contains scRNA-seq data of about 30,000 cells isolated from *Arabidopsis* roots, including roots from heat-treated seedlings. GSE122687 contains scRNA-seq data generated using the drop-seq method from 10 libraries of root cells (~12,000) isolated from *Arabidopsis* plants cultured with (3 libraries) or without (7 libraries) sucrose. The scRNA-seq data of GSE123013 and GSE123818 were generated from not only wild-type *Arabidopsis* plants but also root mutants. The number of cells is 11,030 and 5862 for GSE123013 and GSE123818, respectively. The raw data of the fifth scRNA-seq experiment (PRJNA517021) were downloaded from the NCBI SRA database. The Cellranger software was used to obtain cell expression data by mapping scRNA-seq reads to the TAIR10 genome (https://www.arabidopsis.org/ accessed on 29 August 2021). The expression matrix of 8000 cells was finally obtained.

### 2.2. Data Integration and Sampling

After obtaining the expression matrix of the samples, we performed a preliminary filter on the data using a low stringency, i.e., a gene was considered as expressed if it was detected in at least 3 cells and a cell was retained in the analysis if it had at least 200 expressed genes. The integration (CCA and anchors) method in Seurat V3 was used to integrate the data. Considering the large number of datasets, we adopted the ‘Reference-based’ integration method. A sample with a cell number greater than 5000 was used as a reference, with the remainder designated ‘query’ datasets. After integrating, a total of 56,903 cells were used for downstream analysis. In order to investigate the influence of cell numbers on the analysis of single-cell data, we randomly sampled the integrated data (set.seed = 1234) with 8 different sizes of cell numbers: 500, 1000, 3000, 5000, 10,000, 20,000, 30,000, or 40,000. Each was considered as a subsample. These 8 subsamples plus the originally integrated data from 56,093 cells were compared.

### 2.3. Determination of Significant PC Number

In order to determine the number of significant PCs of the subsamples with different cell numbers, we focused on the first 100 PCs of each subsample (130 PCs for the integrated data) and used the JackStraw function in Seurat V3 to identify ‘significant’ PCs as those that have a strong enrichment of low *p*-value features. When the *p*-values of the first occurrence of PCs were greater than 0.05, the subsequent PCs with *p*-values lower than 0.05 were no longer considered. The Elbow method that plots a ranking of principle components based on the percentage of variance explained by 100 PCs was performed to determine an inflection point. The top 30 PCs of each of the 8 randomly generated subsamples were also used for correlation analysis with the top 30 PCs of the sample containing all 56,903 cells.

### 2.4. Cell Clustering and Assignment of Clusters to Known Cell Types

A total of 2000 genes were used for the clustering analysis. Clusters were identified using the Seurat ‘FindClusters’ function with ‘resolution = 1.0′. The data structures and cell trajectories were separately visualized and explored by PCA, t-SNE, and UMAP methods (both dims = 30). We mainly used the reported marker genes of *Arabidopsis* root cells to accurately assign the cell cluster to a cell type (Supplementary Materials Table S1) and used the Spearman’s correlation coefficient analysis with bulk transcriptome data for verification. In addition, the Index of Cell Identity (ICI) scores were also used for cell type determination and confirmation. ICI score evaluates the expression of hundreds of genes to calculate the likelihood that a single cell belongs to a specific *Arabidopsis* root cell type. In the ICI algorithm, the bootstrap replacement method (1000 iterations) was used to estimate the probability associated with cell type assignment, and the Benjamini–Hochberg method was used to adjust the score. In addition, we further analyzed cluster similarity based on the agreement indices calculated using the clues R-package.

### 2.5. Identifying High-Specificity Genes

Based on the results of cluster annotation, we identified the differentially expressed genes using the FindAllMarker (only.pos = TRUE, min.pct = 0.25, logfc.threshold = 0.25) function in Seurat V3 for the identified cell types. For ROC analysis of DEGs, we also applied the FindAllMarker function by setting the parameter test.use = “roc”.

### 2.6. Pseudotime Analysis

The subsamples with different cell numbers were randomly generated from the integrated dataset that had been analyzed using Seurat V3 by standardization and normalization; so, when performing pseudotime analysis using monocle2, we skipped the process of data standardization. In order to explore the cell differentiation path from the root meristem cell to other cell types, we used monocle (2.14.0) to construct the trajectory using two types of cells (meristem and root hair cell) with different sizes (i.e., cell numbers) of randomly sampled cells. We extracted the subset of the integrated data with cell type information from Seurat object. We set the distribution of expression matrix as “uninormal” when creating CellDataSet object to skip standardization, then ran ‘reduceDimension’ (set norm_method = “none”, pseudo_expr = 0, reduction_method = “DDRTree”) using variance genes coming from the results of Seurat ‘FindVariableFeatures’ to reduce data into low dimensions without further normalization. Based on the expression data in the lower dimension space, we used ‘orderCells’ to identify cell states and constructed cell differential trajectory in pseudotime.

### 2.7. Statistical Analyses for Estimation of Cluster Similarity

#### 2.7.1. Rand Index

The similarity of two clusters in the same dataset can be evaluated by Rand Index (RI) based on the number of pairs in each cluster. If the two clusters are identical, RI equals to 1. If the two clusters are completely different, or if one cluster contains a single element, RI equals to 0.

#### 2.7.2. Morey and Agresti’s Adjusted Rand Index (MA)

The purpose of MA is to overcome the inflation problem caused by RI’s inherent opportunity allocation. Although the value of RI is between 0 and 1, the value of MA ranges from −1 to 1, where negative values indicate independent labels and positive values indicate similar clusters.

#### 2.7.3. Hubert and Arabie’s Adjusted Rand Index (HA)

HA is used to detect and modify the problem in the expected Rand index value of MA.

#### 2.7.4. Fowlkes and Mallows Index (FM)

FM is the geometric mean of precision and recall, and ranges from 0 to 1. A higher FM value indicates a higher similarity between clusters.

#### 2.7.5. Jaccard Index (JI)

JI is another measurement of cluster similarity. The index value ranges from 0 to 1, where a higher JI value reflects a higher cluster similarity.

## 3. Results

### 3.1. Overview of 1244 Reported Single-Cell Transcriptome Studies

**Figure 1:**
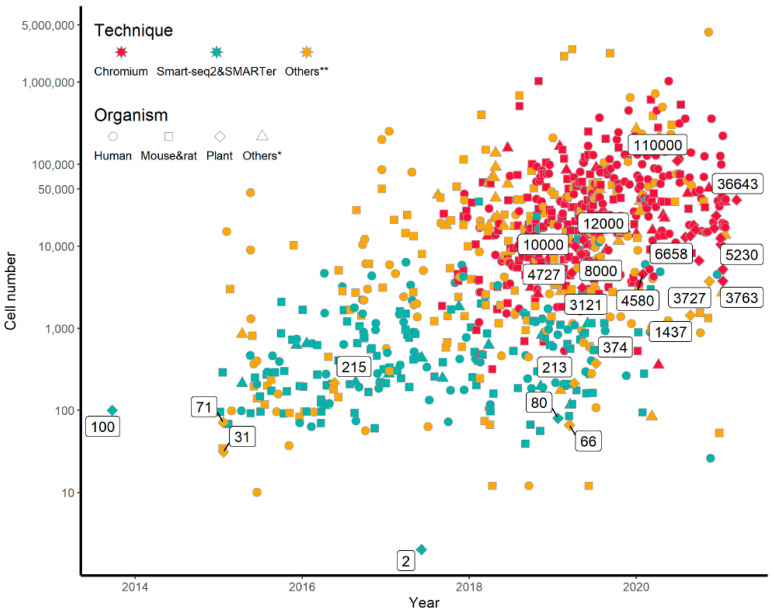
Distribution of sample sizes (i.e., cell numbers) in the recent 1244 studies on single-cell RNA profiling. Technique means the methodology applied in profiling single-cell gene expression. The numbers in the round boxes mean cell number in plant single-cell researches. Distribution of the 1244 studies in different species are also shown in Supplementary Materials Table S2.

A comprehensive comparison of the cell numbers used in published scRNA-seq studies and their outcomes will help us understand current scRNA-seq status and design future scRNA-seq experiments. To this end, we collected and analyzed 1244 published scRNA-seq studies that were collected in the single-cell studies database [31] (http://www.nxn.se/single-cell-studies/ accessed on 29 August 2021) or published articles on scRNA-seq that are not included in the database, including 518 human-related studies, 525 mice/rat-related studies, 30 studies on plants, and 173 studies on other species (Supplementary Materials Table S2; Figure 1). Apparently, most scRNA-seq studies were carried out in humans and model animals. The cell numbers and the scRNA-seq method used in each study were plotted against the year of the study published (Figure 1). It is clear that the majority of the studies published before 2018 used hundreds to thousands of cells and the Smart-seq2 technology. Since releasing the Chromium platform in 2016, 10× Genomics dominated scRNA-seq studies with about 252 papers published after 2018, each using more than 10,000 cells. Notably, some of the studies that used millions of cells were mainly on data mining or database construction. 

Due to the difficulties involved in the preparation of single cells in plants, scRNA-seq study in plants is far behind those in human and animals with respect to both the number of studies and the cell numbers used in each study (Supplementary Materials Table S3). The two early plant scRNA-seq studies sequenced 31 and 215 single cells using CEL-seq and Smart-seq2, respectively [17,18]. Application of the 10× Genomics technology in plants increased the number of cells sequenced to thousands and even ten thousands [24,25,26,27,28] (Supplementary Materials Table S3). The cell types identified in these studies significantly increased compared with the scRNA-seq studies with hundreds or less cells. Among the five scRNA-seq studies published in 2019 on *Arabidopsis* root cells, it seems that more cell types were identified when more input cells were used. For example, Jean-Baptiste et al. (2019) analyzed 3121 cells and identified 5 cell types, including endodermis, cortex, nonhair cell, stele, and hair cell. Shulse et al. (2019) analyzed 12,000 cells and classified them into 17 cell types; however, the study could not assign a distinct cell cluster to quiescent center (QC) cells, probably due to their most central localization in roots and naturally being the least among all cell types in roots. Surprisingly, Denyer et al. (2019) successfully identified QC cells with only ~5000 cells. Comparing the parameters used in cell clustering in these two studies [24,27], we noticed that only 35 principal components (PCs) were used in the Shulse’s study, while 50 PCs were applied in the Denyer’s study. As high-throughput single-cell sequencing data is high-dimensional due to differential expression of numerous genes in a large number of different type of cells, reducing the dimensionality of scRNA-seq data is required for effective data processing and analysis. To achieve that, usually only a certain number of significant PCs are adopted to represent the main difference in dataset. Therefore, the number of PCs selected is expected to be one of the most important parameters affecting subsequent analyses, such as cell clustering, biomarker identification, and trajectory analysis. All of these are main focuses of researches using single-cell RNA profiling and the topics of this study. Next, we investigated the effects of the input cell numbers on significant PC numbers, cell clustering, identification of DEGs, and cell trajectory inference.

### 3.2. Effect of Cell Numbers on Significant PC Numbers

The single-cell transcriptomic data of *A. thaliana* root cells reported in the five studies, which used thousands or more cells (Supplementary Materials Table S3; for more details, see Materials and Methods), were used to study the effect of sample size on single-cell RNA profiling. In order to obtain unbiased subsamples with different cell numbers and to avoid the batch effect between the original samples, we filtered and integrated the original five datasets into a single dataset containing 56,903 cells. Eight subsamples, with sample sizes of 500, 1000, 3000, 5000, 10,000, 20,000, 30,000, or 40,000 cells, were extracted through random sampling of the total 56,903 cells. The 8 subsamples together with the sample containing all 56,903 cells were used in the following analyses.

**Figure 2:**
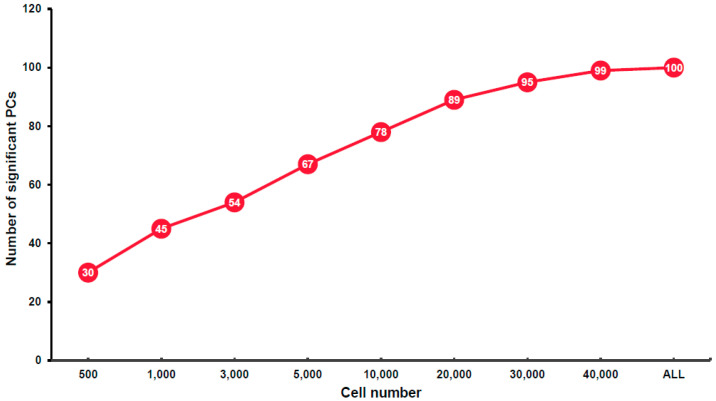
Significant principal component (PC) numbers in the nine subsamples with different cell numbers.

The PC values of each subsample with different cell numbers were analyzed firstly using the Elbow method (Supplementary Materials Figure S1A). In the Elbow plot, the elbow of the curve, or the inflection point, is used to determine the number of PCs that can be used to describe the variation of the dataset. Based on this analysis, no significant difference was observed among the 9 subsamples/integrated-sample (hereafter subsample for simplicity) regarding the inflection point that seems to be between 25 and 30 in each subsample. We then used JackStraw plot to visualize and to identify the number of significant PCs that are defined based on *P*-value (Supplementary Materials Figure S1B). Using a *p*-value < 0.05 as the criterion for a significant PC, we found that the number of significant PCs increases along with the increase in cell number (Figure 2), despite the increase slowing down when the cell numbers reach 30,000. It indicates that when the cell number reaches a certain level, the power of detecting variations among different cells plateaus. The significant PC number is 30 in the subsample with 500 cells based on JackStraw plot, and 30 was also considered an appropriate PC number based on the Elbow method. Based on this result and also for the reason of comparability of the subsequent analyses, for each subsample, we selected the top 30 PCs in the following analyses. To confirm whether 30 was high enough to characterize the integrated data with 56,903 cells, we compared the cell clustering results with 30, 50, and 100 significant PCs and found that, under the same resolution, the number of cell clusters did not change significantly. When the PC number was 30, 50, and 100, the identified cell clusters was 35, 37, and 37, respectively (Supplementary Figure S2A–C), suggesting that the top 50 significant PCs are sufficient to represent most of the variations existing in the subsample with 56,903 cells.

### 3.3. Effect of Cell Numbers on Cell Clustering

**Figure 3:**
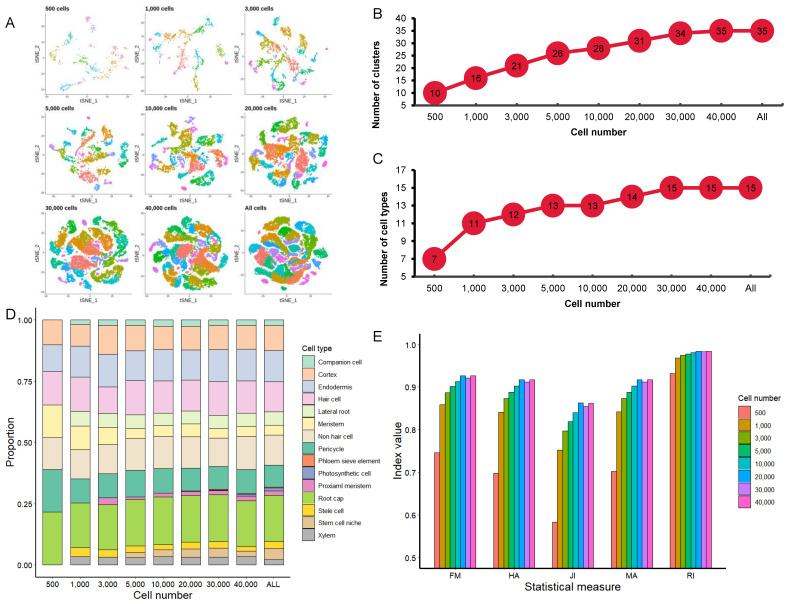
Effect of cell numbers on cell clustering. (**A**) tSNE maps of the nine subsamples with different cell numbers. (**B**) Changes of the number of cell clusters with the increase in cell numbers. (**C**) Changes of the annotated cell types with the increase in cell numbers. (**D**) Composition of cell types annotated in different subsamples. (**E**) The scores of the five statistical measures for evaluating cluster similarity of different sample size. The five indices are Rand index (RI), Morey and Agrest’s adjusted Rand index (MA), Hubert and Arabie’s adjusted Rand index (HA), Fowlkes and Mallows index (FM), and Jaccard index (JI).

We then performed clustering analysis on different subsamples using a PC number of 30. Under the same resolution (res = 1), the tSNE (t-distributed Stochastic Neighbor Embedding) graphs were generated to visualize cell clustering (Figure 3A). The number of cell clusters, to some extent, represents the number of potential cell types in a tissue. When the cell numbers increased from 500 to 40,000, the number of cell clusters increased from 10 to 35 (Figure 3A,B).

We further analyzed the effect of different cell numbers on identifying specific cell types by assigning individual clusters to known root cell types based on marker genes (Supplementary Materials Table S1). We examined the expression of about 200 marker genes that were reported in the five *Arabidopsis* root scRNA-seq studies mentioned above. In order to improve the annotation of marker genes, single-cell RNA profiles were also compared to the reported bulked RNA profiles of different root cell types, which were isolated by using green fluorescent protein (GFP) labeling technology. In addition, we also calculated the index of cell identity (ICI) score for each cell to assign the appropriate cell type. The number of cell types identified increased from 7 to 11 when the cell numbers increased from 500 to 1000, but only 4 more cell types were identified when the cell numbers increased from 1000 to 30,000, and no more new cell types were identified when the cell number was further increased (Figure 3C). The 7 cell types identified in the subsample with 500 cells are the most common cell types in *Arabidopsis* roots, including root cap, pericycle, hair cell, nonhair cell, meristem, endodermis, and cortex. The 4 additional cell types identified in the subsample with 1000 cells are xylem, stele cell, lateral root, and companion cell. The proximal meristem and stem cell niche were identified when the cell numbers were increased to 3000 and 5000, respectively. Phloem sieve element, which forms sieve tube of phloem for transportation of nutrients and is located at the center of *Arabidopsis* roots, was separated by using the subsample with 20,000 cells. However, as a rare cell type in *Arabidopsis* roots, the photosynthetic cell was only detectable with much larger cell numbers (>30,000) (Figure 3D). We also analyzed the correlation between the top 30 PCs identified in each of the 8 subsamples with that of the integrated sample with 56,903 cells. Between the subsample with 500 cells and the integrated-sample with 56,903 cells, only a few major PCs were highly correlated, consistent with the observation that the common cell types in *Arabidopsis* roots could be distinguished by only a few top significant PCs. Importantly, the PC correlation increased as the number of cells increased (Supplementary Materials Figure S3).

In addition, five statistical measurements—Rand index (RI), Morey and Agrest’s adjusted Rand index (MA), Hubert and Arabie’s adjusted Rand index (HA), Fowlkes and Mallows index (FM), and Jaccard index (JI)—were selected to assess the reliability of the clustering (Figure 3E). The cluster information of each subsample was compared against the information of the All data set. All five measurements were the lowest in the subsample with 500 cells, and without a significant difference in the three subsamples with 20,000 to 40,000 cells, indicating that the reliability will have little improvement by using more than 20,000 cells. This shows that as the number of cells increases, the reliability of clustering will also increase, but when the number of cells reaches a certain level, its increasing trend will slow down or even pause.

### 3.4. Effect of Cell Numbers on Identification of Differentially Expressed Genes

As a traditional transcriptome analysis, scRNA-seq-based identification of differentially expressed genes (DEGs) can explore the unique expression patterns of different cell types in the studied tissues. We used DEGs identified in five cell types (hair cell, nonhair cell, root cap, cortex, and endodermis) to compare the effect of sample size or cell numbers. The number of DEGs (592–708) identified in each subsample was not highly correlated with the number of cells used in each subsample, as the number of DEGs were similar in the subsamples with 3000 to 56,903 cells (Supplementary Materials Figure S4A). In total, 784 nonredundant DEGs were identified in the 9 subsamples. Approximately two thirds (521 or 67%) existed in all subsamples, 47 (6%) were found in 8 subsamples except the subsample with 500 cells, and 30 (4%) were found in 7 subsamples except the subsamples with 500 or 1000 cells. These results indicate that 77% of the DEGs could be successfully identified with only 3000 cells and 96% with 20,000 cells (Supplementary Figure S4A). The number of DEGs unique to each subsample declined as the cell numbers increased. In order to explore the reliability of the DEGs, we performed ROC analysis for the DEGs common in all subsamples and unique to each subsample. It was obvious that the median AUC value of the common DEGs (>0.8) was much higher than that of unique DEGs (~0.65) (Supplementary Materials Figure S4B).

Meanwhile, we compared the DEGs identified in the 5 cell types with the marker genes of these cell types identified using the traditional bulk transcriptome analysis [32]. As we selected 2000 genes from the samples for data integration, we thus firstly checked their overlapping in the 9 subsamples. The overlapping genes were then compared with the DEGs identified in the 5 cell types to identify a new set of overlapping genes, which we found depended on cell types and sample size. For example, few overlapping genes were found in nonhair cells, which were almost constant in all subsamples, but in cortex cells, the number of overlapping genes increased in the subsamples with 500 to 3000 cells and remained almost the same in subsamples with 3000 to 56,903 cells (Supplementary Materials Figure S4C). 

### 3.5. Effect of Cell Numbers on Cell Trajectory Inference

**Figure 4:**
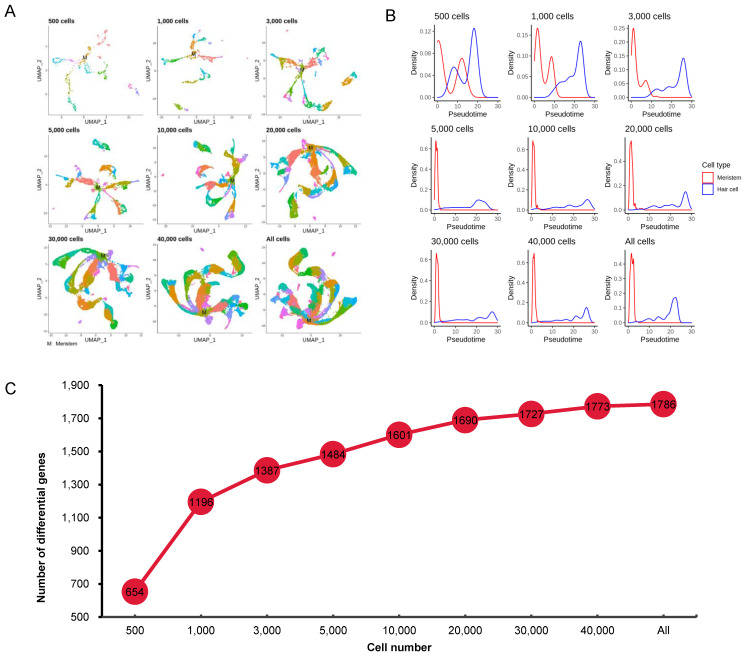
Effect of cell numbers on cell trajectory inference. (**A**) The UMAP map of nine subsamples with different cell numbers. (**B**) Density distribution map of meristem and hair cells on the pseudotime axis in the nine subsamples with different cell numbers. (**C**) The number of differentially expressed genes that change as a function of pseudotime.

In order to explore the effect of sample size on interpretation of cell growth and development by scRNA-seq, we drew a map for each subsample by UMAP (Uniform Manifold Approximation and Projection) [33] (Figure 4A), which showed that the meristem cell had a clear trend towards multiple cell types. As the number of cells increases (such as to 20,000 cells), this trend became more obvious. We further selected two cell types (meristem cell and hair cell) to investigate the impact of cell numbers on cell trajectory analysis. 

Although the pseudotimes showing in the density map (Figure 4B) could not be used for direct comparison, with the help of the distribution of meristem cells, we could make a reasonable judgment on the pseudotime of root hair cells. For meristem cells, a relatively accurate pseudotime could not be estimated with a sample size of 3000 cells or less; however, the estimated pseudotime, which was concentrated in the 0–5 interval, did not change in the subsamples with 5000 or more cells. Compared with the pseudotime of meristem cells, the pseudotime of hair cells also could not be estimated in the subsample with only 500 cells, as the root hair cells in this subsample were concentrated in the area overlapping with the meristem cells, but a relatively distinct pseudotime could be estimated in the subsamples with 1000 and 3000 cells. This analysis also revealed that development of root hair cells lasted for a much longer time than that of meristem cells. 

In addition, trajectory inference is crucial for discovering genes that are associated with lineages in the trajectory to clarify potential biological processes. In the trajectory-based differential expression analysis, the number of DEGs increased with the increase in sample size. However, when the sample size reached a certain level (e.g., 20,000 cells), the growth rate of DEGs was significantly reduced (Figure 4C).

## 4. Discussion

scRNA-seq has been widely adopted to uncover the complex gene expression patterns of different cell types in tissues. With the development of single-cell-related technologies—for instance, droplet-based microfluidic systems—the number of cells used in single-cell research has increased dramatically. However, it is still a challenge to isolate a large number of single cells from some tissues, particularly plant tissues. In addition, given the cost involved in scRNA-seq, it is important to find a good balance between the expense and the outcome of an experiment. It is thus essential to know the effect of sample size on the results of scRNA-seq. Our results presented here, together with previously published results, indicate that sample size has a significant impact on the results of single-cell analysis. Suner (2019) [34] found that the clustering performance of the currently available methods used in scRNA-seq data analysis depends largely on the sample size and the complexity of the biological materials. However, a better result cannot be achieved by simply increasing the number of cells used in sequencing. For example, Bhaduri A et al. (2018) [35] found that most biologically interpretable cell types in the database with 1.3 million cells can be distinguished by 50,000 randomly selected cells. 

We reasoned that a suitable cell number required for profiling certain tissues to provide helpful information for follow-up investigations can be estimated by analyzing the published real data from a sufficient number of cells. We therefore combined the scRNA-seq data of *Arabidopsis* root cells from five independent studies and analyzed the effect of sample size on cell clustering, identification of DEGs, and rare cell types. We noticed that some rare cell types in *Arabidopsis* roots—for example, phloem sieve element and photosynthetic cell—could be identified by using ~56,000 cells, but some, such as QC cells, could not be found with the same number of cells. Increasing the number of cells may increase the likelihood of finding QC cells; however, development of a customized algorithm specifically for the identification of rare cell types may be a better alternative [36,37,38]. On the other hand, as the outcomes of single-cell experiments are affected by many experimental factors such as enzymatic hydrolysis efficiency and cell capture efficiency, designing experiments specifically for the targeted rare cell types, such as tagging the target cells with GFP marker, should be another cost-effective option.

## 5. Conclusions

This study implies that there is a saturated cell number for the power of plant scRNA profiling. We propose that ~20,000 (or 10,000–30,000) cells are enough for profiling Arabidopsis root cells, although whether its applicability to other Arabidopsis tissues and other plant species is yet to be confirmed by future scRNA-seq studies on diverse plant species and tissues. Nevertheless, our results provide a general guide for optimizing sample size to be used in plant scRNA-seq studies.

## Data Availability

The datasets described or used in this study are available in the NCBI Sequence Read Archive under BioProject accession number GSE121619, GSE122687, GSE123013, GSE123818, and PRJNA517021.

## Supplementary Materials

The following are available online at https://www.mdpi.com/article/10.3390/cimb43030119/s1, Figure S1: The Elbow plots (A) and the JackStraw plots (B) of the nine sub-samples with different cell numbers, Figure S2: The effect of 30 (A), 50 (B), and 100 (C) significant PCs on the cell clustering results, Figure S3: Correlation analysis between the top 30 PCs identified in each of the eight sub-samples with that identified in the original integrated dataset with 56,903 cells, Figure S4: Effect of cell numbers on identification of differentially expressed genes, Table S1: Marker genes, Table S2: Summary of the studies on single-cell RNA profiling analyzed in this study, Table S3: Comparison of the cell numbers estimated by HowManyCell and SCOPIT.
