# Supplementary material for: Effects of Sample Size on Plant Single-Cell RNA Profiling
Source: Curr Issues Mol Biol. 2021 Oct 20;43(3):1685–97. doi: 10.3390/cimb43030119 (PMC8929096; doi:10.3390/cimb43030119)
Supplement: Supplementary file 1 [file cimb-43-00119-s001.zip › Supplementary figures and tables/Table S2.pdf]

Table S2. Summary of the studies on single-cell RNA profiling analyzed in this study. See Fig. 1 for the number of cells used in each study.

**Organism**	**Number of papers**	**Median number of cells sampled**
Human	518	32,648
Mouse and rat	525	49,167
Plant	28	4,582
Others*	173	27,840
**Technique platform**		
Chromium (10x Genomics)	247	43,031
Smart-seq2	97	4,447
Others**	341	47,142

* include zebrafish, *C. elegans*, drosophila, yeast, *Schmidtea mediterranea*, frog, pig, plasmodium, planarian, sea squirt, sea urchin, slime mold, macaque, *Nematostella vectensis* and dictyostelium.

** include CEL-seq2, Drop-seq, Microwell-seq, SCRB-seq, MARS-seq, STRT-seq2, Fluidigm C1 and BD-Rhapsody.
