# Supplementary material for: Effects of Sample Size on Plant Single-Cell RNA Profiling
Source: Curr Issues Mol Biol. 2021 Oct 20;43(3):1685–97. doi: 10.3390/cimb43030119 (PMC8929096; doi:10.3390/cimb43030119)
Supplement: Supplementary file 1 [file cimb-43-00119-s001.zip › Supplementary figures and tables/Table S3.pdf]

Table S3. Summary of single-cell RNA profiling studies in plants. "/" means unable to get detail informations.

Species	Tissue/Site	Protocol	No.Cells	Reference
Solanum lycopersicum	vasculature/lateral root	10x Genomics	960	Omary et al., 2020
	shoot apex	10x Genomics	/	Tian et al., 2020
Arabidopsis thaliana	vegetative shoot apex	10x Genomics	36,643	Zhang et al., 2021
	seed	/	1,437	Picard et al., 2020
	female gametic cells	/	/	Song et al., 2020
	root	10x Genomics	110,000	Shahan et al., 2020
		10x Genomics	15,918	Wendrich et al., 2020
		10x Genomics	4,727	Denyer et al., 2019
		10x Genomics	3,121	Jean-Baptiste et al., 2019
		10x Genomics	>10,000	Ryu et al., 2019
		10x Genomics	>12,000	Shulse et al., 2019
		Drop-seq	374	Turco et al., 2019
		10x Genomics	8,000	Zhang et al., 2019
		Smart-seq2	/	Efroni et al., 2016
		/	31	Efroni et al., 2015
		10x Genomics(scRNA+scATAC)	10,548	Farmer et al., 2021
	root;leaf;flower;seed;silique;stem	Nanowell-based approach	3,727	Sunaga-Franze et al., 2020
	root/embryo	10x Genomics	1,762	Long et al., 2021
	cotyledon	10x Genomics	12,844	Liu et al., 2020
	whole aerial tissue or first true leaves	10x Genomics & Smart-seq2	18,000	Lopez-Anido et al., 2020
	lateral root	10x Genomics	6,658	Gala et al., 2020
	sperm cells	Smart-seq2	80	Misra et al., 2019
	leaf	10x Genomics	5,230	Kim et al., 2021

Oryza sativa	root	10x Genomics	23,532	Liu et al., 2020
	the aerial part of rice seedlings	10x Genomics	4,580	Wang et al., 2020
Physcomitrella patens	leaf	/	66	Kubo et al., 2019
Zea mays	shoot apical meristems	10x Genomics & CEL-seq2	13,377	Satterlee et al., 2020
	anther	CEL-seq2	213	Nelms et al., 2019
	ears	10x Genomics	12,525	Xu et al., 2021
	leaf	10x Genomics	3,763	Bezrutczyk et al., 2021
