# Supplementary figures and images for: Effects of Sample Size on Plant Single-Cell RNA Profiling
Source: Curr Issues Mol Biol. 2021 Oct 20;43(3):1685–97. doi: 10.3390/cimb43030119 (PMC8929096; doi:10.3390/cimb43030119)

## Slide 1
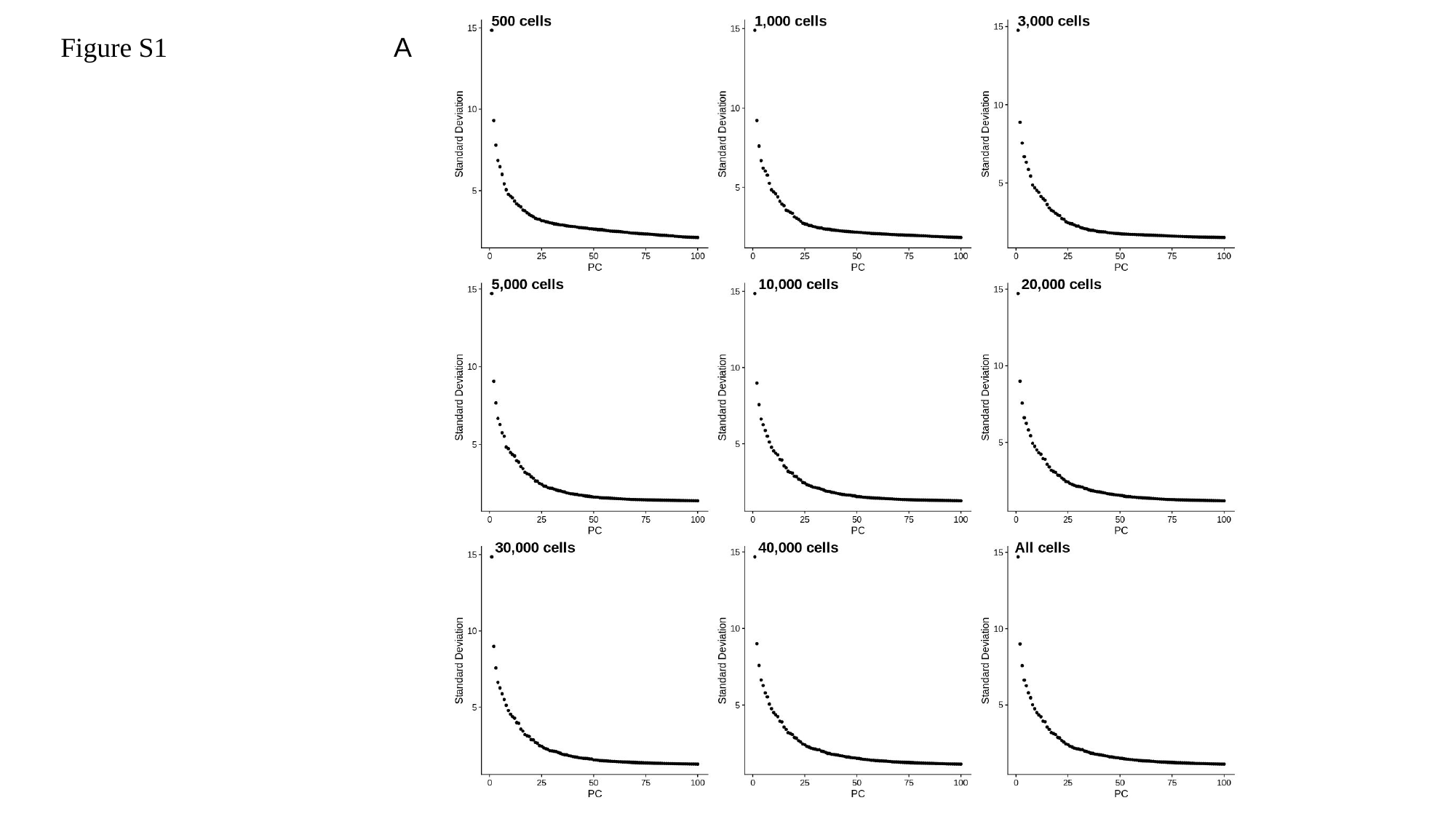

Figure S1
A

## Slide 2
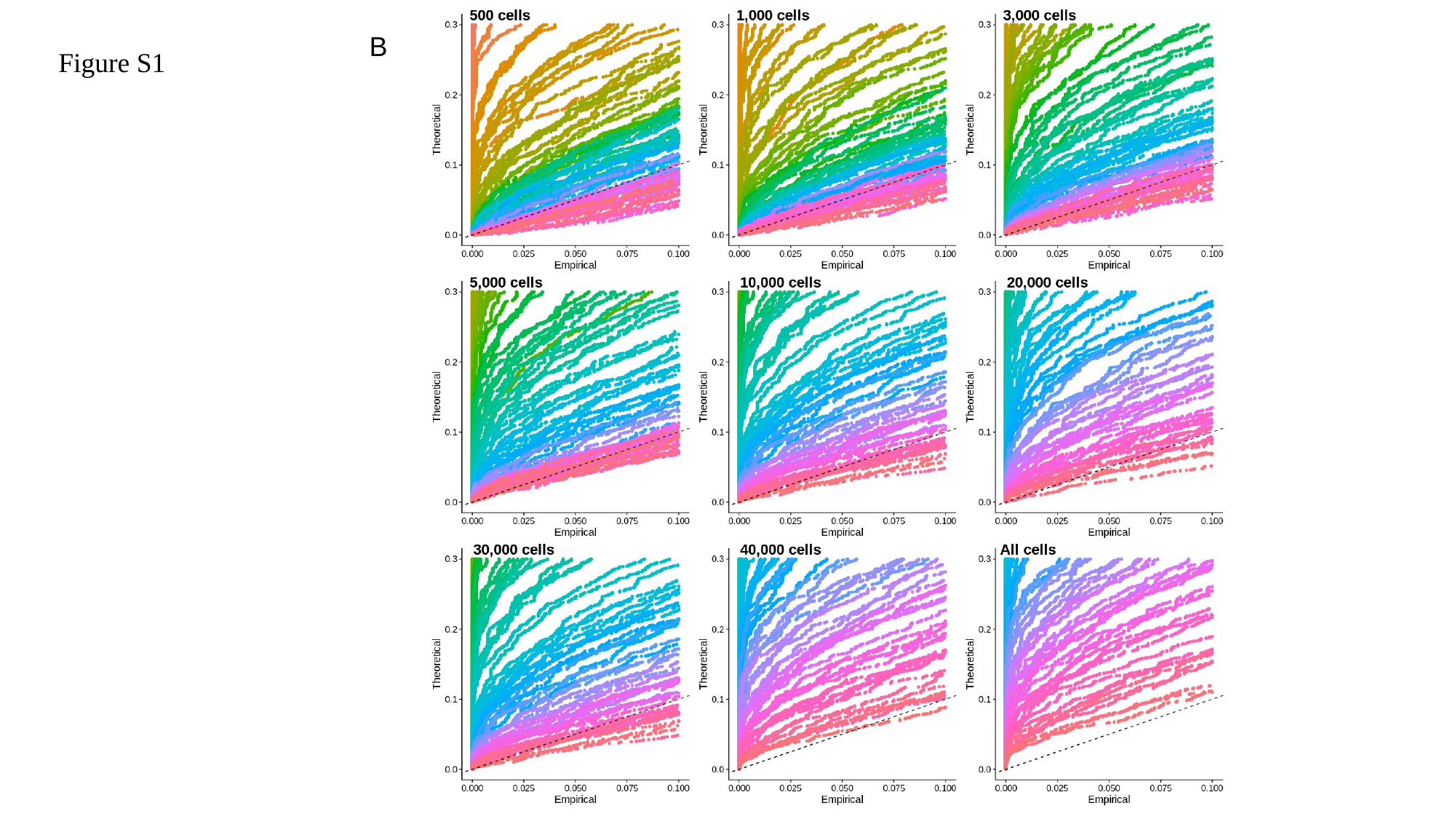

B
Figure S1

Supplement: Supplementary file 1 [file cimb-43-00119-s001.zip › Supplementary figures and tables/Figure S1.pptx]

## Slide 1
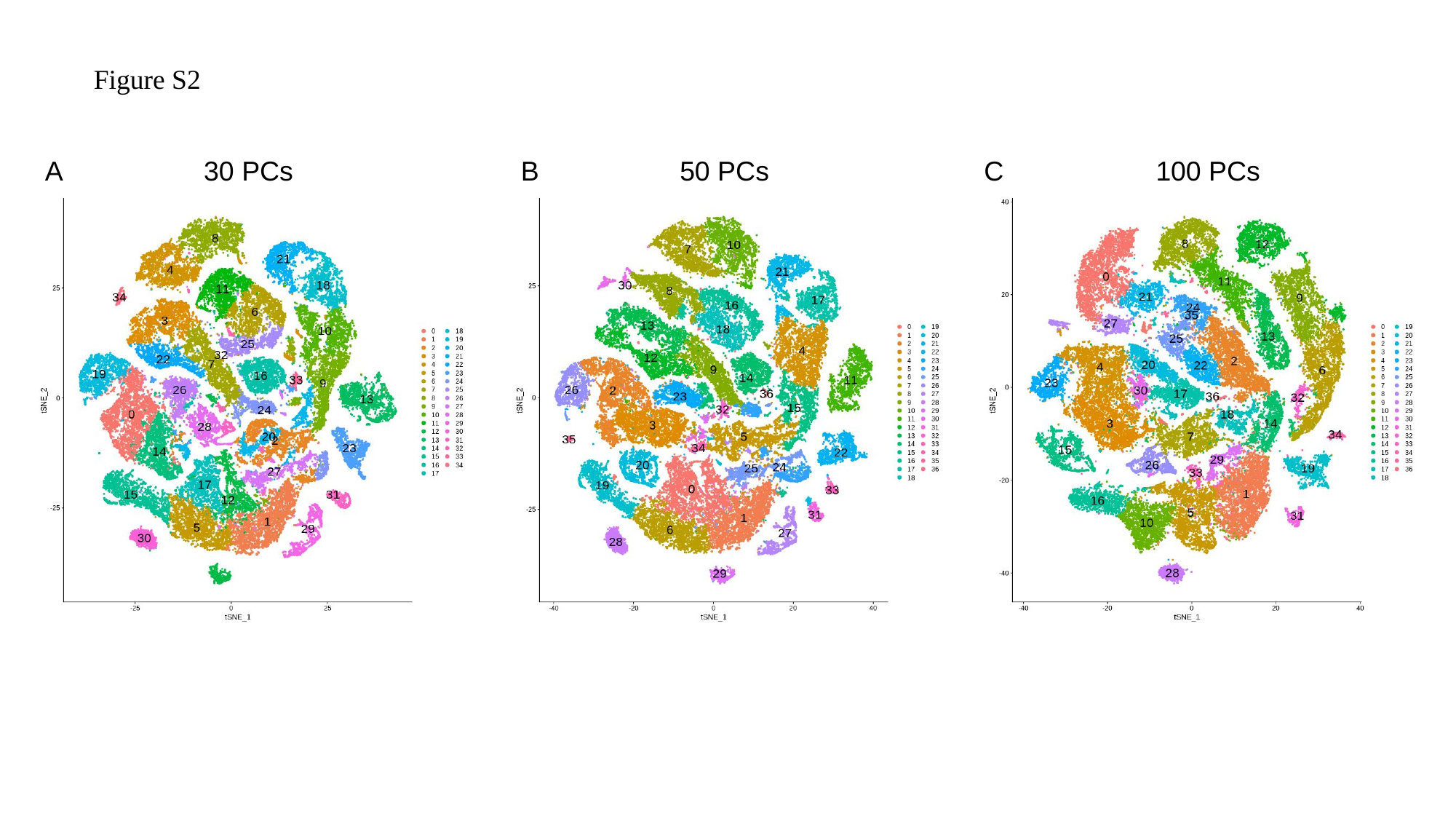

Figure S2
A
B
C
30 PCs
50 PCs
100 PCs

Supplement: Supplementary file 1 [file cimb-43-00119-s001.zip › Supplementary figures and tables/Figure S2.pptx]

## Slide 1
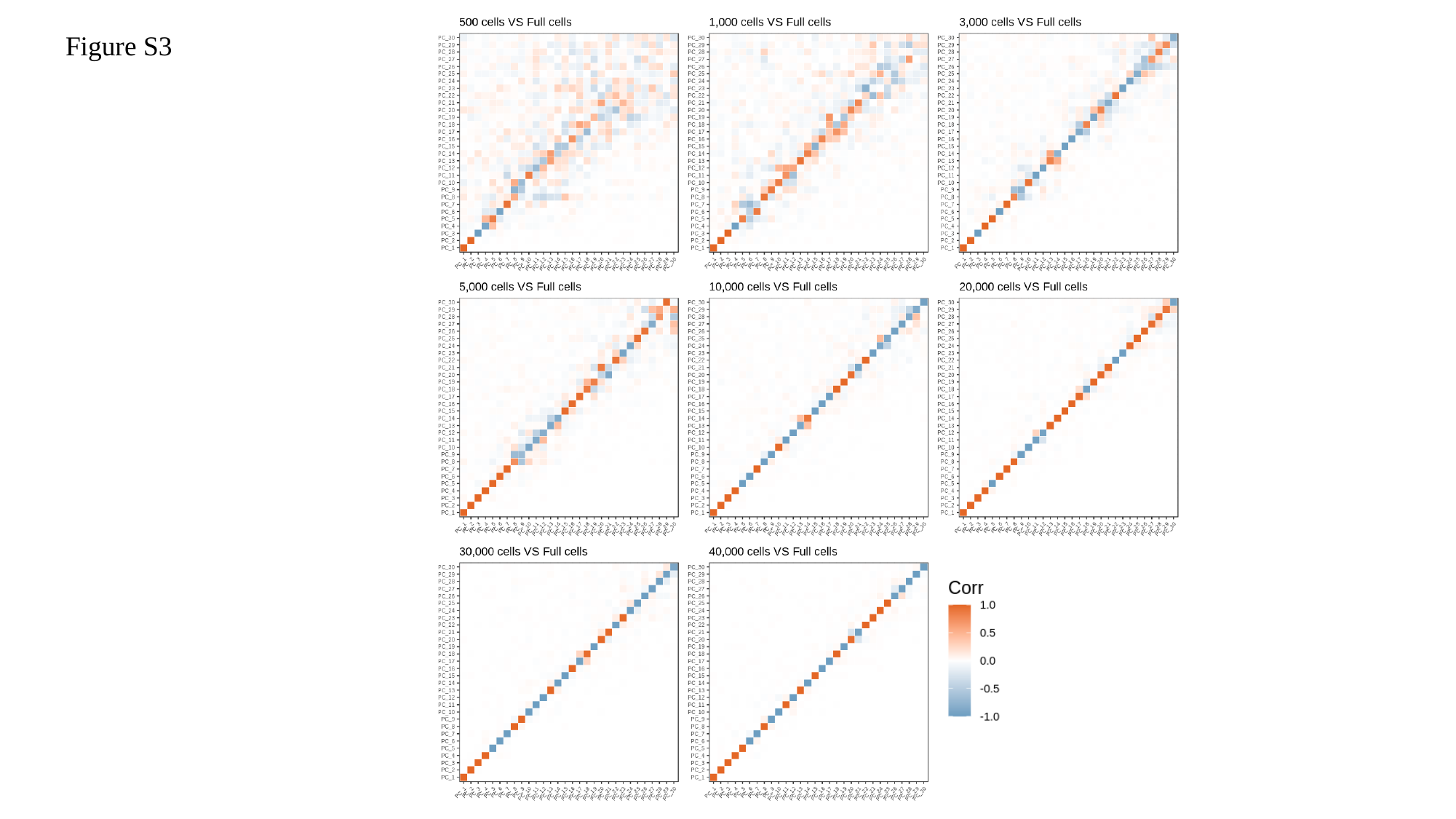

Figure S3

Supplement: Supplementary file 1 [file cimb-43-00119-s001.zip › Supplementary figures and tables/Figure S3.pptx]

## Slide 1
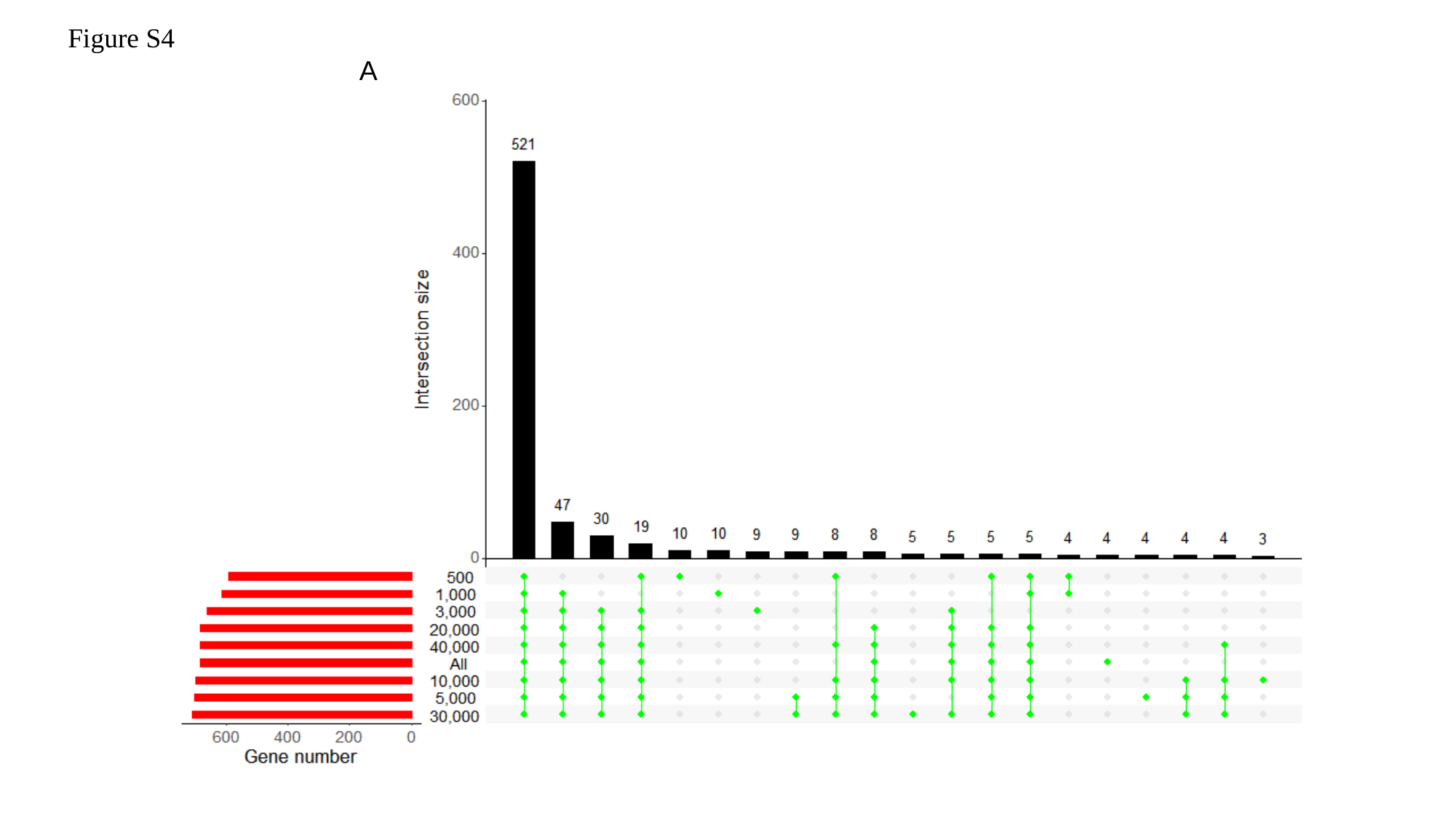

Figure S4
A

## Slide 2
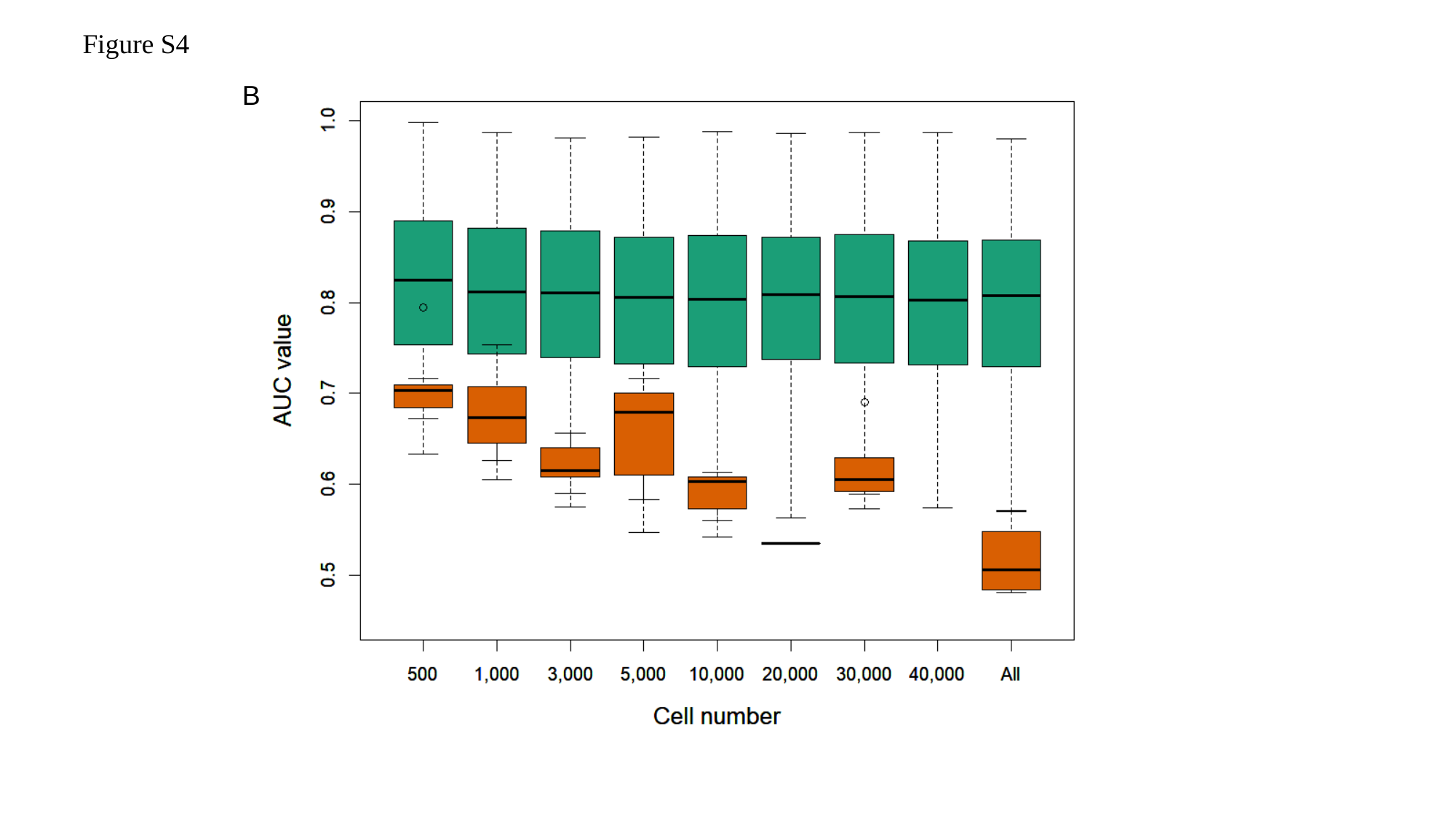

Figure S4
B

## Slide 3
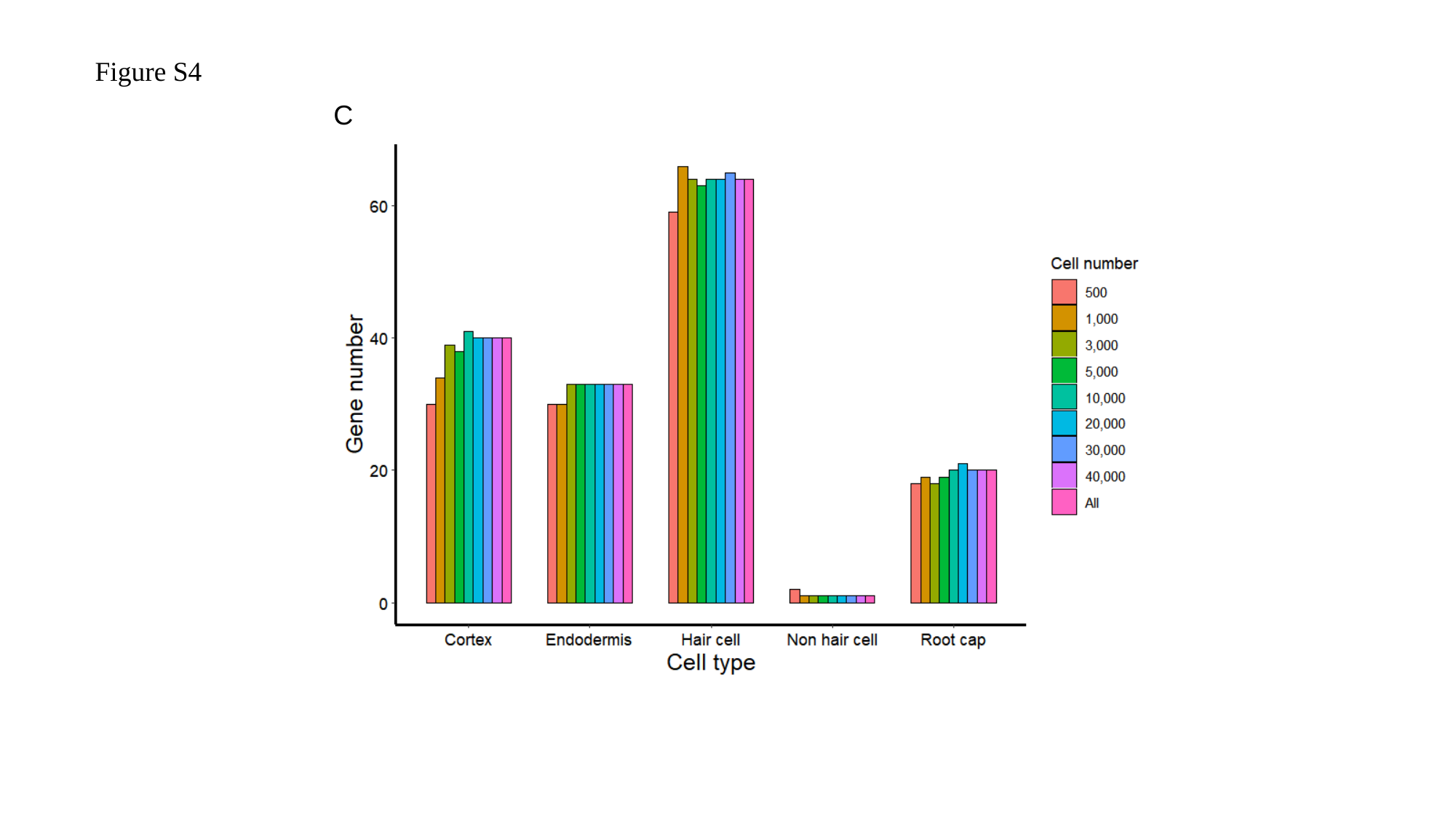

Figure S4
C

Supplement: Supplementary file 1 [file cimb-43-00119-s001.zip › Supplementary figures and tables/Figure S4.pptx]
